# Executive Function in Preschool Children with Congenital Heart Disease and Controls: The Role of a Cognitively Stimulating Home Environment

**DOI:** 10.1016/j.jpeds.2023.113897

**Published:** 2024-01-01

**Authors:** Andrew T. M. Chew, Alexandra F. Bonthrone, Arezoo Alford, Christopher Kelly, Kuberan Pushparajah, Alexia Egloff, Joseph V. Hajnal, John Simpson, Mary Rutherford, A. David Edwards, Chiara Nosarti, Serena J. Counsell

**Affiliations:** 1 Centre for the Developing Brain, School of Biomedical Engineering and Imaging Sciences, King’s College London, London, United Kingdom; 2 Paediatric Cardiology Department, Evelina London Children’s Healthcare, London, United Kingdom; 3 Department of Child and Adolescent Psychiatry, Institute of Psychiatry, Psychology and Neuroscience, King’s College London, London, United Kingdom

## Abstract

**Objective:**

To assess the relationships between (1) environmental and demographic factors and executive function (EF) in preschool children with congenital heart disease (CHD) and controls and (2) clinical and surgical risk factors and EF in preschool children with CHD.

**Study design:**

At 4-6 years of age, parents of children with CHD (n = 51) and controls (n = 124) completed the Behavior Rating Inventory of Executive Function, Preschool Version questionnaire and the Cognitively Stimulating Parenting Scale (CSPS). Multivariable general linear modeling assessed the relationship between Behavior Rating Inventory of Executive Function, Preschool Version composite scores (Inhibitory Self-Control Index [ISCI], Flexibility Index [FI], and Emergent Metacognition Index [EMI]) and group (CHD/control), sex, age at assessment, gestational age, Index of Multiple Deprivation, and CSPS scores. The relationships between CHD type, surgical factors, and brain magnetic resonance imaging injury rating and ISCI, FI, and EMI scores were assessed.

**Results:**

The presence of CHD, age at assessment, sex, and Index of Multiple Deprivation were not associated with EF scores. Lower gestational age was associated with greater ISCI and FI scores, and age at assessment was associated with lower FI scores. Group significantly moderated the relationship between CSPS and EF, such that CSPS significantly predicted EF in children with CHD (ISCI: *P* = .0004; FI: *P* = .0015; EMI: *P* = .0004) but not controls (ISCI: *P* = .2727; FI: *P* = .6185; EMI: *P* = .3332). There were no significant relationships between EF scores and surgical factors, CHD type, or brain magnetic resonance imaging injury rating.

**Conclusions:**

Supporting parents to provide a cognitively stimulating home environment may improve EF in children with CHD. The home and parenting environment should be considered when designing intervention studies aimed at improving EF in this patient group.

Congenital heart disease (CHD) affects almost 1% of births in the United Kingdom and is the most common congenital malformation. Improvements in antenatal diagnosis, cardiac surgery, and perioperative care mean that most infants born with CHD now survive.^1^ However, children and adults with CHD are at increased risk of adverse neurodevelopmental sequalae, including impaired motor, language, and cognitive development.^2,3^ One important higher-order cognitive function is executive function (EF). EF could be regarded as an umbrella term for a range of cognitive processes, including attention, working memory, planning, inhibition, self-monitoring, self-regulation, and initiation.^4,5^ EF components have been related to early mathematics and reading abilities, and children with greater EF are better prepared for schooling.^6–8^

Previous studies have suggested that school-aged children, adolescents, and adults with CHD have an increased prevalence of EF difficulties.^9–12^ In addition, perinatal, clinical,^13–16^ and family environmental factors^17^ reportedly influence neurodevelopmental outcomes in individuals with CHD. We have shown previously that a more stimulating home environment is associated with better cognitive function in toddlers with CHD.^18^

To date, there have been few studies assessing EF in preschool children with CHD. The aims of this study were to assess (1) the relationships between environmental and demographic factors and EF in preschool children with CHD and controls (a “heart healthy” community sample) and (2) the relationships between surgical risk factors (days to surgery, time on bypass, days on intensive care) and EF in preschool children with CHD. We hypothesized that (1) EF scores would be lower in children with CHD compared with those from the community sample, (2) greater socioeconomic status and a more cognitively stimulating environment would be associated with greater EF scores in both groups, and (3) increased exposure to perisurgical risk factors would be associated with lower EF in the CHD sample.

## Methods

The study was approved by the National Research Ethics Committee (19/LO/0451). In accordance with the Declaration of Helsinki, informed written parental consent was obtained before questionnaire data were collected. (Please see the Appendix for further details of the parental consent process; available at www.jpeds.com).

Inclusion criteria for the CHD sample were children with critical or serious CHD who underwent neonatal magnetic resonance imaging (MRI) of the brain as part of the Congenital Heart Imaging Project (REC: 07/H0707/105) between September 2014 and May 2018 and who had surgery or intervention by cardiac catheterization within the first year of life. Critical CHD was defined as hypoplastic left heart syndrome, transposition of the great arteries (TGA), pulmonary atresia with intact ventricular septum, interruption of the aortic arch, and all infants requiring surgery within the first 28 days of life with the following conditions: coarctation of the aorta, aortic valve stenosis, pulmonary valve stenosis; tetralogy of Fallot (TOF), pulmonary atresia with ventricular septal defect, and total anomalous pulmonary venous connection. Serious CHD was defined as any cardiac lesion not defined as critical that requires cardiac catheterization or surgery before 1 year of age.^19,20^

Inclusion criteria for the control sample were children who participated in the Developing Human Connectome Project (https://www.developingconnectome.org/, REC: 14/LO/1169) and whose parents had consented to be approached for further research studies. We approached parents of participants in the Developing Human Connectome Project who were the same age (projected age at assessment) as participants with CHD. Exclusion criteria for both groups were children born before 31 weeks of gestational age.

EF was assessed using the Behavior Rating Inventory of Executive Function, Preschool Version (BRIEF-P),^21^ which is a 63-item parent-completed questionnaire. The BRIEF-P has been validated and is widely used in research follow-up,^22,23^ in various communities,^24,25^ and in different clinical settings^26,27^ to assess the presence and severity of executive dysfunction in day-to-day situations. It contains 5 clinical scales that denote different aspects of executive functioning: Inhibit, Shift, Emotional Control, Working Memory, and Plan/Organize. (Further details of the BRIEF-P scales can be found in the Appendix; available at www.jpeds.com.)

Three indexes can be derived from the BRIEF-P: Inhibitory Self-Control (ISCI), Flexibility (FI), and Emergent Metacognition (EMI). This study uses raw scores to determine ISCI (derived from inhibition and emotional control raw scores), FI (derived from the shift and emotional control raw scores), and EMI (derived from the working memory and plan/organize raw scores) as summary measures of EF. The ISCI represents a child’s ability to modulate responses, actions, emotions, and behavior via appropriate inhibitory control. The FI represents a child’s ability to switch flexibly between actions, responses, emotions, and behavior. The EMI represents a child’s ability to initiate, plan, organize, execute, and sustain future-oriented problem-solving.^21^ The raw scores of all 3 indexes were used for analysis in order to account for the full range of variation in these scales. Greater scores represent poorer EF.

The Cognitively Stimulating Parenting Scale (CSPS),^18,28,29^ completed by parents, is a 28-item questionnaire adaptation of the Home Observation for Measurement of the Environment Inventory.^30^ The CSPS assesses the availability and variety of experiences that promote cognitive stimulation at home and in the family. This includes availability of educational toys, parental interactions such as teaching words or reading stories, and cognitively stimulating activities such as family excursions or trips.^18^

Index of multiple deprivation (IMD) was derived from the residence postcode when follow-up questionnaires were posted to parents. IMD is a composite measure of socioeconomic status in England, combining information from 7 domains to produce an overall relative measure of deprivation. Residential postcode was used to calculate IMD from the 2015 data release and reported as quintiles and percentile ranks. (http://imd-by-postcode.opendatacommunities.org/; Accessed December 13, 2022).

The number of days from birth to corrective or final palliative surgery, time on bypass during surgery, and number of days on the intensive care unit postsurgery were obtained from the hospital records. In children who underwent more than 1 surgery, days on intensive care unit and time on bypass were summed across procedures.

MRI of the brain was obtained before surgery in children with CHD and in the neonatal period for control children. MRI pulse sequence parameters are described in Kelly et al.^20^ Images were reported by a perinatal neuroradiologist. Lesions were classified as focal arterial ischemic stroke, white matter injury (WMI), cerebellar hemorrhage, or intraventricular hemorrhage, as we have reported previously.^20^ WMI was classified into normal, mild, moderate, or severe.^31^ Overall, each baby was categorized into 1 of 4 brain injury groups: normal, mild (intraventricular hemorrhage, and/or cerebellar hemorrhage ≤2 mm, and/or mild WMI), moderate (cerebellar hemorrhage >2 mm and/or moderate WMI), and severe brain injury (focal arterial ischemic stroke and/or severe WMI) as described previously.^20^

All data were analyzed in SPSS, version 28 (IBM Corp). Data were assessed for normality using histograms, Shapiro–Wilk tests, skewness, and kurtosis values. ISCI, FI, and EMI scores; CSPS; gestational age at birth; and age at assessment were not compatible with a normal distribution.

Multivariable general linear modeling was undertaken to assess the relationship between ISCI, FI, and EMI scores; group (CHD or control) sex; age of assessment; gestational age at birth; IMD; and CSPS scores. Results were corrected for multiple tests using false discovery rate correction^32^ and *P* < .05 after correction was considered significant. In a post-hoc analysis, moderated multiple regression analyses were conducted to examine the role of group (CHD or control) in the relationship between CSPS and EF outcome measures, controlling for age, sex, GA, IMD, and brain MRI injury rating (BIR) using the PROCESS 4.2 macro.^33^

The relationship between CHD type (1) abnormal mixing (ie, TGA, truncus arteriosus), (2) left sided-lesions (ie, coarctation of the aorta), and (3) right-sided lesions (ie, TOF, pulmonary atresia or stenosis, tricuspid atresia), surgical factors, and BIR (moderate and severe BIR were combined) and ISCI, FI, and EMI scores were assessed. Age at assessment, sex, gestational age at birth, and IMD and CSPS scores were included as covariates in the analysis. Kruskal–Wallis tests were used for categorical data. Associations between continuous variables and ISCI, FI, and EMI scores were tested using nonparametric partial correlations accounting for the covariates. Results were corrected for multiple tests (3 EF measures) using false discovery rate correction^32^ and *P* < .05 after correction was considered significant. All secondary analyses were repeated after removing children with CHD who had a confirmed or suspected genetic disorder.

## Results

Eighty parents of children with CHD agreed to receive questionnaires, and 61 were returned (76% return rate). Five children were excluded, as they were born at less than 31 weeks of gestational age, and 5 children did not have cardiac surgery. Data from 51 children with CHD were included in the analysis. Five children in the CHD sample had confirmed or suspected genetic abnormality (2 children had CHARGE [coloboma, heart defects, atresia choanae—also known as choanal atresia, growth retardation, genital abnormalities, and ear abnormalities] syndrome, 2 had 22q11 deletion, and 1 child had a suspected but not confirmed genetic abnormality). The relationship between cognitive outcome and CSPS scores at 2 years in 43 of these children was described previously.^18^

**Figure 1:**
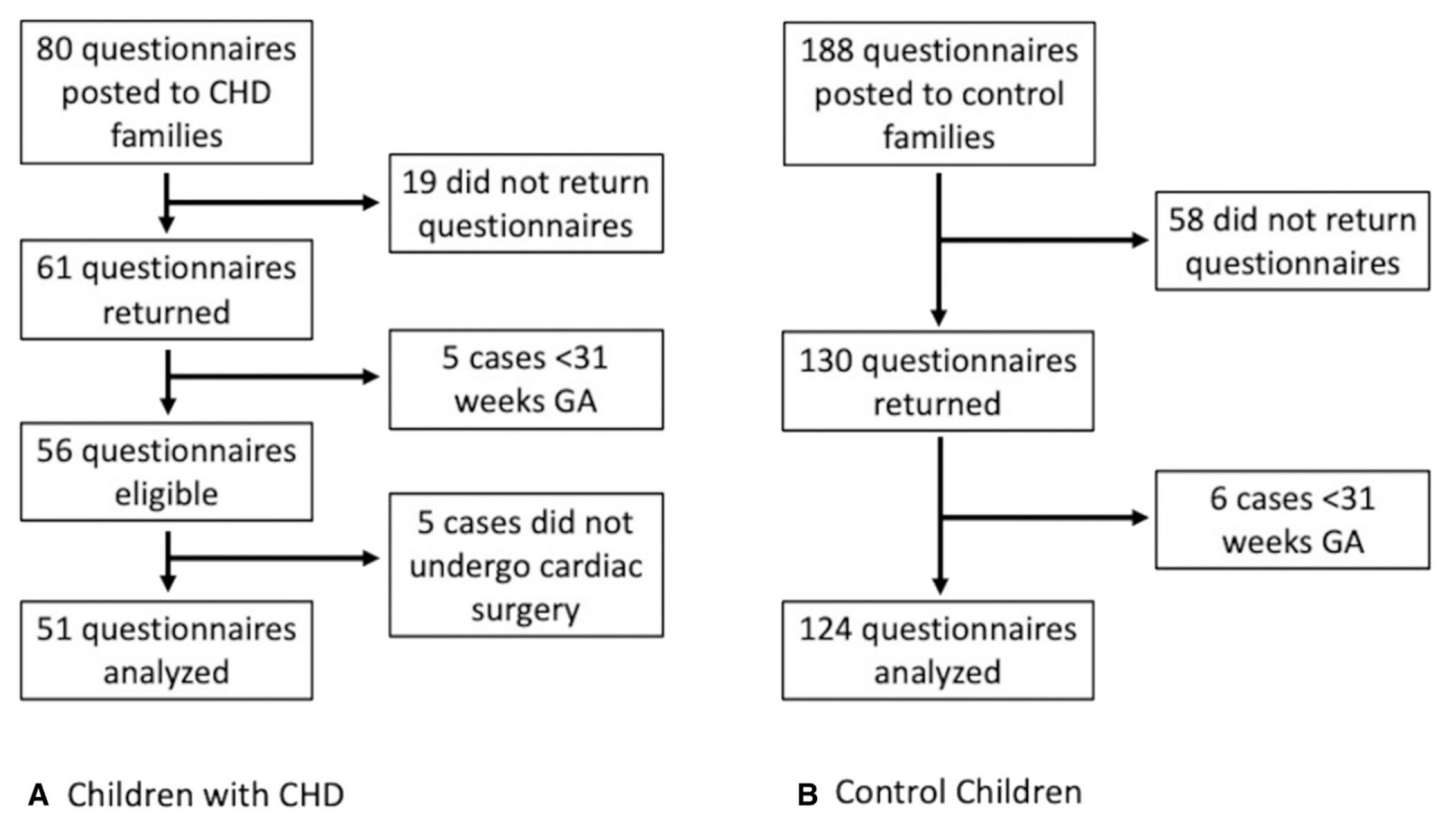
Flow chart of participant recruitment for both CHD and control groups. *CHD*, congenital heart disease.

In total, 188 parents of control children agreed to receive questionnaires and 130 were returned (69% return rate). Six children were born at less than 31 weeks of gestational age. The final control sample size was 124 children. Sixteen children in the control group did not complete neonatal neuroimaging. Figure 1 shows details of participant recruitment.

The primary cardiac diagnoses and clinical characteristics of the children with CHD are summarized in Table I. Table II shows the demographic, environmental, and MRI findings of the brain for the children. Children with CHD were born at a younger gestational age (*P* < .001) and had lower CSPS scores (*P* = .003). There was no difference between CHD and controls in sex distribution (*P* = .573), IMD (*P* = .856), BIR (*P* = .149), or age at assessment (*P* = .773).

Table III shows BRIEF-P raw scores and T-scores in children with CHD and controls. Multivariable general linear modeling identified no significant relationships between presence of CHD, age at assessment, sex, IMD, and EF scores. Gestational age at birth was significantly negatively correlated with ISCI and FI scores, and age at assessment was significantly negatively associated with FI. CSPS was significantly negatively correlated with ISCI, FI, and EMI scores (Table IV). When BIR was added to the multivariable analysis (n = 108 controls), the relationships between all 3 EF scores and CSPS remained significant. ISCI and FI remained significantly negatively correlated with gestational age at birth and age at assessment remained significantly negatively correlated with FI (Table V).

**Figure 2:**
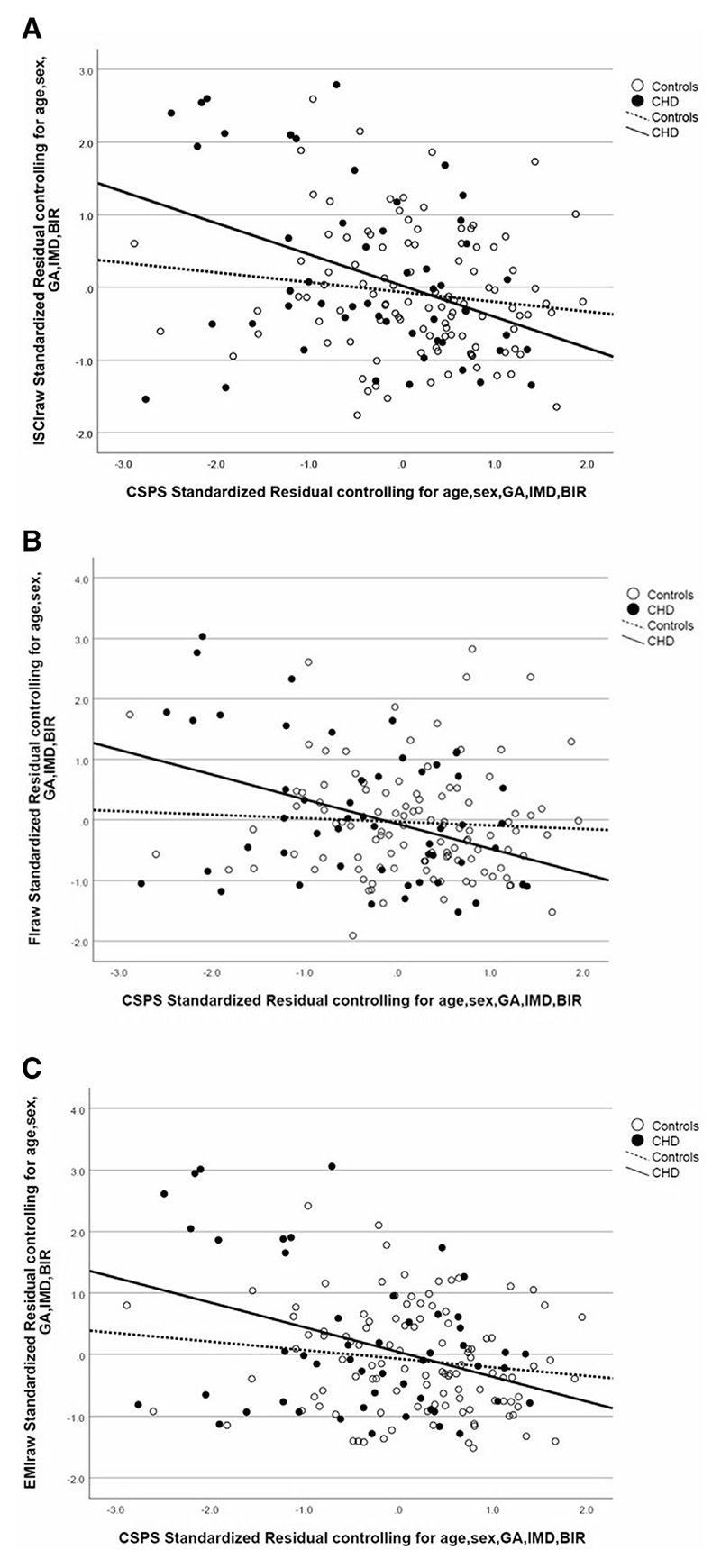
Cognitively stimulating parenting scale scores significantly predicted **A**, ISCI scores in the CHD group (*P* = .0004, R^2^ = 0.148) but not in controls (*P* = .273, R^2^ = 0.02); **B**, FI scores in the CHD group (*P* = .0015, R^2^ = 0.149) but not in controls (*P* = .619, R^2^ = 0.003); and **C**, EMI scores in the CHD group (*P* = .0004, R^2^ = 0.129) but not in controls (*P* = .333; R^2^ = 0.021). All analyses were co-varied for age at assessment, gestational age at birth, sex, IMD, and BIR.

There was a significant interaction of group (CHD, control) and CSPS scores in predicting all 3 EF outcomes (ISCI: *P* = .045; FI: *P* = .034; EMI: *P* = .038) in childhood such that CSPS scores significantly predicted all 3 EF outcomes in children with CHD (ISCI: *P* = .0004; FI: *P* = .0015; EMI: *P* = .0004) but did not in controls (ISCI: *P* = .2727; FI: *P* = .6185; EMI: *P* = .3332) (Figure 2, A-C). There was no significant interaction of group (CHD, control) and gestational age in predicting any of the 3 EF outcomes (ISCI: *P* = .2866; FI: *P* = .1118; EMI, *P* = .4579).

There were no significant relationships between BRIEF-P scores and surgical factors, CHD type, or brain MRI injury rating (Table VI). The results were largely unchanged when the 5 children with confirmed or suspected genetic disorders were excluded. See the Appendix (available at www.jpeds.com) for further details.

## Discussion

Our results did not demonstrate any significant differences in day-to-day EF scores between children with CHD and controls when controlling for relevant demographic and environmental factors. In addition, when assessing only the children with CHD, we did not identify any significant relationships between EF and perisurgical clinical factors, CHD type, or brain MRI injury rating. However, we identified a significant interaction between group (CHD, controls) and a cognitively stimulating home environment for various aspects of EF: ISCI, FI, and EMI. CSPS scores significantly predicted all 3 EF outcomes in the CHD group but did not in controls.

This study provides further evidence of the role of a cognitively stimulating home environment in cognitive performance and executive function in at-risk groups of children. We have shown previously that a more cognitively stimulating home environment is associated with greater cognitive scores on the Bayley Scales of Infant and Toddler Development, Third Edition, at 22 months in toddlers with CHD,^18^ and this study highlights that the benefits of a cognitively stimulating home environment persist until preschool age. In children born preterm at 4-7 years, cognitively stimulating parenting was associated with decreased developmental psychopathology and executive dysfunction^29^ and, in both children born at term and preterm, cognitively stimulating parenting supported academic resilience in middle childhood.^28^ There is a great deal of interest in understanding how enriched environments affect outcome in children at risk of neurodevelopmental disorders^34,35^ but, to date, there are very few studies that include controls.^36^ Although the mechanisms are not clear, our findings suggest a cognitively stimulating environment increases child resilience, leading to improved neurodevelopmental outcomes in at-risk groups of children.

While our finding that group (CHD or control) did not influence EF scores is supported by a recent study in preschool children at 5 years with CHD, which showed that parent-rated EF scores did not differ from normative means,^37^ other studies have identified impaired EF at older ages in individuals with CHD. However recent meta-analyses have shown considerable between-study heterogeneity, including differences in study designs in EF assessments, and outcomes in this population.^38,39^

In a mixed CHD case series of 8- to 16-year-old children and adolescents, the BRIEF metacognition index was raised relative to test norms and above the 90th centile in 24% of cases.^40^ At school age (9 years), a greater proportion of children with CHD who required surgery in the first year of life had elevated metacognition and behavior regulation scores compared with controls. In this study, aortic obstruction rather than univentricular heart was associated with worse EF scores.^9^ In adolescents, a case–control study found that rates of EF impairment, assessed using, were almost twice as high for the CHD sample compared with the control sample, and distinct EF profiles were observed between CHD types.^10^ In another study in adolescents with TGA who underwent the arterial switch operation as infants, the Global Executive Composite scores on the BRIEF questionnaires as completed by parents and teachers were significantly worse than the expected population mean.^11^ Similarly, in adolescents with TOF, both parent- and teacher-reported Global Executive Composite scores were greater than the expected population mean, with a greater number of scores being of clinical concern. It is worth noting that the adolescent self-reports were similar to normative values, suggesting the adolescents do not consider themselves to have EF impairments.^41^ In adults, a large study in the United Kingdom showed individuals with CHD exhibited deficits in problem-solving, attention, and verbal fluency compared with normative data.^12^ However, a recent study in a heterogenous sample of adults with CHD showed that self-reported and informant-reported EF scores were similar to normative data and were similar between different CHD types.^42^

To date, the number of studies comparing EF in preschool children with CHD to control children are limited. Using face-to-face assessments to measure cognitive inhibition and flexibility, behavioral inhibition and working memory in children with TGA and controls aged 4-6 years, Calderon et al demonstrated worse EF scores in almost all tests in the TGA group (n = 45) compared with controls (n = 45).^43^ However, demographic characteristics of the control group were not compared with the TGA sample, and so it is possible that, in addition to CHD, demographic and environmental factors may play a role in these differences. Prenatal diagnosis of TGA was significantly related to cognitive flexibility, when controlling for family SES and parental education level.^43^ Prenatal diagnosis enables planning for perinatal care, including appropriately trained staff and medication in the delivery room,^44^ and is associated with improved hemodynamic stability.^45–47^ All but one child in our study was diagnosed antenatally and so this may, at least in part, contribute to our findings of no differences in EF between control and CHD groups.

In our sample of mixed CHD diagnoses, we did not observe any association between CHD type and EF. However, our sample did not include any children with hypoplastic left heart syndrome, who are at greater risk of EF impairments in later childhood and adolescence.^10^ Risk factors for neurodevelopmental impairments in children with CHD include lower gestational age at birth,^48,49^ longer times to surgery, time on cardiopulmonary bypass, and length of hospital stay.^13–16^ We did not observe a relationship between perinatal clinical or surgical factors and EF outcomes in our CHD group. However, despite excluding children born at less than 31 weeks, lower gestational age at birth was associated with worse ISCI and FI scores. Preterm birth is a recognized risk factor for poorer EF.^50^ Previous studies have shown that preschool children born of extremely low birth weight have poorer inhibition and working memory, whereas those born late preterm also showed impaired working memory abilities.^51^ Another study of children born very preterm (assessed between 4 and 12 years) identified difficulties in inhibition, working memory, and planning when compared with term-born controls.^52^ Even as young adults, those born very preterm continue to show impaired inhibition and mental flexibility when compared with controls.^53^ Our findings could be studied in relation to timing of brain maturational changes occurring during the last trimester of gestation, when most infants who are preterm are born. Corticostriatal pathways, centrally implicated in the establishment of FI and ISCI,^54^ reach their maximal growth phase from 24 weeks of gestational age to term.^55^ Metacognition is predominantly anchored in prefrontal brain regions,^56^ which reach maximal growth in adolescence.^57^ Such timing of prefrontal cortex maturation could possibly explain why we did not observe a relationship between gestational age and EM in this study.

We also found that younger age of assessment was associated with greater FI scores. Cognitive flexibility begins developing after the age of 3 years, and there continues to be rapid advances between the ages of 4-6 years.^58^ In contrast, we did not observe significant associations between age at assessment and the 2 other BRIEF-P indices, ISCI and EMI. We interpret these results in relation to timing of development of emerging metacognition and inhibitory self-control, as the age range of our study participants was relatively narrow. Although development of metacognition begins in early childhood,^59^ significant improvements in children’s capacity to reflect and efficiently evaluate their performance becomes established later, between 5.5 and 7.5 years.^60^ Improvements in inhibitory control refine throughout childhood and adolescence^61^ and are particularly pronounced later in childhood and the preadolescent years (age 8-11 years), in association with frontostriatal brain maturation.^62^

We found no effect of brain injury on EF scores. However, there were few infants with moderate-to-severe brain injury on MRI in our sample. The role of neonatal WMI and later EF is not clear as, to our knowledge, there have been no other studies assessing the role of neonatal brain injury on pre-school EF in CHD. Although moderate-severe neonatal WMI has been associated with reduced full-scale IQ at 6 years,^63^ a meta-analysis reported that there was no consistent relationship between preoperative brain injury and neurodevelopmental outcome in this study group.^64^

Our study has some limitations. Our CHD sample size is not large, although it is similar to other studies at this age. In addition, children in our CHD sample were born at a younger gestational age than controls. This is characteristic of CHD cohorts, particularly those with a prenatal diagnosis,^65^ and we controlled for gestational age at birth in our multivariable analysis. Intellectual impairment is associated with poorer EF,^66,67^ and we did not include any measure of intelligence in our study. Our data collection commenced in October 2020, when face-to-face assessments were not possible due to the coronavirus disease 2019 pandemic, and so our study design focused on parental questionnaires, which did not require face-to-face assessments. It is not possible, therefore, to determine whether our findings are specific to EF or are part of a global cognitive pattern. In addition, it should be noted that parent-rated assessments of EF may assess different constructs to performance-based measures. In addition, the IMD was used in our analyses as a measure of neighborhood deprivation, and this is based on the participant’s residence postcode. This measure has limited sensitivity and specificity for identifying individuals who are income or employment deprived,^68^ hence the lack of access to a complementary measure reflecting socioeconomic status may represent a further limitation of our study.

This study suggests that impaired EF in survivors of CHD may be mitigated by supporting parents to provide a cognitively stimulating home environment before the child reaches school age, and the home and parenting environment should be considered when designing interventional studies aimed at improving EF in children with CHD. ◼

## Supplementary Material

Appendix

## Figures and Tables

**Table I:** Clinical characteristics of the CHD group

CHD type and clinical characteristics	
Cardiac physiology	n = 51
Cyanotic heart disease, No. (%)	36 (71)
Heart defect causing abnormal mixing of blood	
Transposition of the great arteries, No. (%)	21 (41)
Truncus arteriosus, No. (%)	1 (2)
Left-sided abnormalities of the heart	
Coarctation of the aorta, No. (%)	11 (22)
Right-sided abnormalities of the heart	
Tetralogy of Fallot, No. (%)	11 (22)
Pulmonary stenosis, No. (%)	3 (6)
Pulmonary atresia, No. (%)	3 (6)
Tricuspid atresia, No. (%)	1 (2)
Clinical characteristics	
Days to corrective or palliative surgery, median (IQR)	15 (9-159)
Cumulative minutes on bypass during surgery, median (IQR)	144 (68-187)
Cumulative number of days on ICU postsurgery, median (IQR)	4.0 (3.0-8.0)
Antenatal diagnosis, No. (%)	50 (98)

*ICU*, intensive care unit.

**Table II:** Demographic, environmental, and BIR scores in children with CHD and controls

Characteristics	CHD, n = 51	Controls, n = 124	*P* value
Gestational age at birth, wk, median (IQR)*	38.4 (37.4-38.9)	39.6 (38.1-40.8)	<.001
Sex, male (%)^†^	25 (50)	55 (44)	.573
BIR, No. (%)^† ‡^			.149
None	30 (58.8)	80 (74.1)	
Mild	14 (24.1)	18 (16.7)	
Moderate/severe	7 (13.7)	10 (9.3)	
Age at assessment, mo, median (IQR)*	51.0 (48.5-60.3)	51.0 (48.4-61.0)	.773
IMD quintile, No. (%)^†^			.856
IMD1 (lowest)	7 (14.0)	16 (13.0)	
IMD2	13 (26.0)	42 (33.9)	
IMD3	10 (20.0)	22 (17.7)	
IMD4	11 (20.0)	25 (20.2)	
IMD5 (highest)	10 (20.0)	19 (15.3)	
CSPS, median (IQR)*	36.0 (31.0-39.0)	38.0 (35.0-40.0)	.003

*BIR*, brain MRI injury rating; *CHD*, congenital heart disease; *CPSC*, Cognitively Stimulating Parenting Scale; *IMD*, Index of Multiple Deprivation.

* Mann–Whitney *U* test.

† *χ*^2^.

‡ BIR, n = 108

**Table III:** BRIEF-P raw scores and T scores in children with CHD and controls

	Raw scores			T scores	
BRIEF-P	CHD, n = 51	Controls, n = 124		CHD, n = 51	Controls, n = 124
Inhibitory Self-Control Index (ISCI), median (IQR)	40.0 (35.0-53.0)	38.5 (32.0-45.0)		52.0 (46.0-66.0)	49.5 (42.0-58.0)
Flexibility Index (FI), median (IQR)	31.0 (25.0-38.0)	29.0 (26.0-34.0)		54.0 (44.0-63.0)	48.0 (44.0-57.0)
Emergent Metacognition Index (EMI), median (IQR)	39.0 (32.0-47.0)	37.0 (31.0-44.0)		51.0 (44.0-61.0)	48.5 (41.0-57.0)

**Table IV:** Results of multivariate analysis showing the relationship between BRIEF-P indexes scores and demographic and environmental factors

Multivariate analysis CHD and control children (degrees of freedom = 175)			
Factors	ISCI	FI	EMI
Group (CHD vs control)	F = 0.672, *P* = .532	F = 0.060, *P* = .908	F = 1.308, *P* = .381
Age assessed	F = 3.084, *P* = .182	F = 7.344, *P* = .032*	F = 1.038, *P* = .429
Gestational age at birth	F = 8.450, *P* = .024*	F = 6.945, *P* = .032*	F = 1.686, *P* = .321
Sex	F = 4.481, *P* = .093	F = 2.049, *P* = .277	F = 2.084, *P* = .277
IMD	F = 0.005, *P* = .991	F = 0.000, *P* = .991	F = 0.587, *P* = .534
CSPS	F = 11.904, *P* < .001*	F = 6.056, *P* = .045*	F = 10.708, *P* = .009*

*P* values after false discovery rate correction.

* *P* < .05.

**Table V:** Results of multivariate general linear modeling showing the relationship between BRIEF-P indexes scores and demographic and environmental factors including BIR rating

Multivariate analysis CHD and control children with neonatal MRI (degrees of freedom = 159)			
Factors	ISCI	FI	EMI
CHD	F = 0.636, *P* = .641	F = 0.009, *P* = .924	F = 0.802, *P* = .641
Age at assessment	F = 2.269, *P* = .201	F = 5.734, *P* = .048*	F = 1.049, *P* = .307
Gestational age at birth	F = 5.622, *P* = .033*	F = 5.213, *P* = .036*	F = 1.110, *P* = .294
Sex	F = 2.291, *P* = .359	F = 0.841, *P* = .361	F = 1.397, *P* = .359
IMD	F = 0.030, *P* = .872	F = 0.026, *P* = .872	F = 0.718, *P* = .472
BIR	F = 2.009, *P* = .198	F = 3.751, *P* = .165	F = 1.674, *P* = .198
CSPS	F = 9.859, *P* = .006*	F = 5.970, *P* = .048*	F = 9.185, *P* = .009*

*P* values after false discovery rate correction.

* *P* < .05.

**Table VI:** Correlations between BRIEF-P Indexes scores and clinical and BIR in CHD cohort, and correlations between surgical data and BRIEF-P Indexes scores covarying for age at assessment, sex, gestational age at birth, IMD, CSPS, and BIR score

Factors	ISCI	FI	EMI
CHD type (abnormal mixing, left-sided lesions, right sided-lesions)	H = 1.379 *(P* = .727)*	H = 1.258 (*P* = .727)	H = 1.666 (*P* = .727)
Time to surgery, d	r(43) = –0.157 (*P* = .727)^†^	r(43) = –0.178 (*P* = .727)	r(43) = –0.118 (*P* = .727)
Time on bypass, min	r(43) = –0.096 (*P* = .727)	r(43) = –0.147 (*P* = .727)	r(43) = –0.012 (*P* = .939)
Days in ICU	r(43) = –0.042 (*P* = .907)	r(43) = –0.015 (*P* = .939)	r(43) = 0.071 (*P* = .801)
BIR	H = 1.996 (*P* = .727)	H = 3.989 (*P* = .727)	H = 1.999 (*P* = .727)

*FI*, Flexibility Index; *EMI*, Emergent Metacognition Index; *ICU*, intensive care unit; *ISCI*, Inhibitory Self-Control Index.

*P* values are false discovery rate corrected.

* H = Kruskal–Wallis statistic.

† r(41) is partial correlation with df = 41.

## Abbreviations

BIR Brain: MRI injury rating
BRIEF-P: Behavior Rating Inventory of Executive Function, Preschool Version
CHD: Congenital heart disease
CSPS: Cognitively Stimulating Parenting Scale
EF: Executive function
EMI: Emergent Metacognition Index
FI: Flexibility Index
IMD: Index of Multiple Deprivation
ISCI: Inhibitory Self-Control Index
MRI: Magnetic resonance imaging
TGA: Transposition of great arteries
TOF: Tetralogy of Fallot
WMI: White matter injury
