# Supplementary material for: Executive Function in Preschool Children with Congenital Heart Disease and Controls: The Role of a Cognitively Stimulating Home Environment
Source: J Pediatr. Author manuscript; Available in PMC 2024 Jul 18. (PMC7616251; doi:10.1016/j.jpeds.2023.113897)
Supplement: Appendix [file EMS197096-supplement-Appendix.docx]

**Appendix**

**Executive function in preschool children with congenital heart disease and controls: the role of a cognitively stimulating home environment.**

**Methods**

**Parental consent process**

1. A developmental pediatrician (ATMC) telephoned parents of children who participated in the CHiP and dHCP projects to enquire whether they would like to participate in this study.
2. The patient information sheet and consent form were emailed/posted to the parents who agreed to be involved in the study.
3. A follow-up phone call was arranged to allow the parents to ask any questions they may have prior to signing the consent form.
4. The BRIEF-P and the Cognitively Stimulating Parenting Scale questionnaires were posted to parents along with a pre-paid return envelope. Parents were asked to return the completed questionnaires and signed consent form.

**BRIEF-P Scales**

The Inhibit scale assesses inhibitory control and impulsivity. This describes the ability to resist impulses and the ability to stop own behaviour at the appropriate time. The Shift scale assesses the ability to move freely from one situation or activity to another as the circumstances require. Key aspects of shifting include the ability to (a) make transitions, (b) tolerate change, (c) problem-solve flexibly, and (d) switch or alternate attention. The Emotional Control scale measures the impact of executive function difficulties on emotional expression and assesses a child’s ability to modulate or control his emotional responses. The Working Memory scale measures “on-line representational memory;” this is the capacity to hold information in mind for the purpose of completing a task, encoding information, or generating goals, plans, and sequential steps to achieving goals. Working memory in young children is essential to sustain problem-solving activities, carry out multistep activities, complete basic mental manipulations, and follow complex instructions. The Plan/Organize scale measures the child’s ability to manage current and future-oriented task demands within the situational context. The scale consists of two task-related components: planning and organization. The plan component relates to the ability to anticipate future events, implement instructions or goals, and develop appropriate steps ahead of time in order to carry out a task or activity.

**RESULTS**

**Secondary analyses excluding children with confirmed or suspected genetic disorders.**

The results were largely unchanged when the 5 children with confirmed or suspected genetic disorders were excluded. Specifically, multivariate general linear modelling showed there was no significant relationships between presence of CHD (ISCI p = 0.488, FI p = 0.965, EMI p =0.488), sex (ISCI p =0.254, FI p = 0.448, EMI p =0.407) , IMD (ISCI p = 0.757, FI p = 0.757, EMI p =0.310) or brain injury rating (ISCI p = 0.233, FI p = 0.129, EMI p =0.310) and EF scores. However, age at assessment was no longer significantly negatively correlated with FI (ISCI p = 0.433, FI p = 0.056, EMI p =0.407), GA was no longer significantly negatively correlated with any EF score (ISCI p =0.071, FI p = 0.129, EMI p =0.448) and CSPS was significantly negatively correlated with ISCI (p <0.001) and EMI (p =0.017) but was no longer significantly correlated with FI (p=0.071) after FDR correction.

The significant interaction of group (CHD, control) and CSPS scores in predicting all three EF outcomes remained (ISCI p=0.005; FI p=0.009; EM: p=0.017). Similar to the whole group analysis, CSPS scores significantly predicted all three EF outcomes in children with CHD (ISCI p<0.001; FI p=0.004; EMI p=0.0002) but did not in controls (ISCI p=0.277; FI p=0.658; EMI p=0.355).

There were no significant relationships between BRIEF-P scores and CHD type (ISCI p=0.183; FI p=0.183; EMI p=0.183), time to surgery (ISCI p=0.997; FI p=0.997; EMI p=0.97), time on bypass (ISCI p=0.770; FI p=0.770; EMI p=0.811), days in ICU (ISCI p=0.997, FI p=0.997, EMI p=0.997), or brain MRI injury rating (ISCI p=0.597; FI p=0.597; EMI p=0.597).
